# Content Analysis of Church Leaders’ Perceptions and Attitudes Toward Dementia

**DOI:** 10.1093/geroni/igaa057.521

**Published:** 2020-12-16

**Authors:** Kimberly Foster, Fayron Epps

**Affiliations:** 1 Georgia State University, Atlanta, Georgia, United States; 2 Emory University, Georgia, United States

## Abstract

Individuals with dementia face many challenges, including the reactions others have to their diagnoses and associated symptoms. Those affected by dementia often seek support from their faith communities and find many of their faith leaders are unable to respond to their needs due to a lack of awareness. In an effort to transform the perception of dementia within the faith community, dementia educational workshops for church leaders were held at three African American churches in Metropolitan Atlanta. As part of the dementia workshop education, an exercise was used to assess attendees’ perceptions and attitudes toward dementia pre and post workshop. One hundred and eight participants took part in this exercise. At the beginning and end of the workshop, participants were asked to write a word or short phrase that came to mind when they thought of dementia. Qualitative content analysis was conducted and 15 codes were extracted and categorized into 3 groups: positive (e.g., supportive); negative (e.g., horrified); and neutral (e.g., information-seeking). Before the workshop, participant responses trended towards negative responses (e.g., fear, loss, damn). After the workshop, participants expressed in writing more neutral and positive phrases and words (e.g., support, hopefulness, caring). The findings indicate that with training, church leaders can change their perceptions and attitudes toward dementia to a more accepting and positive stance. Ultimately, continued education in the faith communities may allow for families affected by dementia to feel comfortable seeking help from their church, a very important resource in their community.

